# Adrenal gland laceration in adult trauma patients without severe concomitant abdominal organ injuries: Incidence, associated factors, and outcomes from a national trauma database study

**DOI:** 10.1097/MD.0000000000041756

**Published:** 2025-03-07

**Authors:** Musaed Rayzah

**Affiliations:** a Department of Surgery, College of Medicine, Majmaah University, Ministry of Education, AL-Majmaah City, Riyadh, Saudi Arabia.

**Keywords:** adrenal gland, injury severity, laceration, mortality, trauma

## Abstract

Adrenal gland lacerations are sporadic but hostile injuries. The purpose of this investigation was to ascertain the prevalence, contributing variables, and outcomes of adrenal gland laceration in adult trauma victims. A retrospective cohort study analyzed data from 649,696 adult patients in the National Trauma Data Bank to compare patient characteristics, injury severity, and outcomes between patients with and without adrenal gland injuries. Multivariate logistic regression was used to control for confounding variables and identify significant associations. The analysis excluded patients with severe liver, spleen, kidney, and gallbladder injuries to focus on the impact of adrenal gland injuries, while minimizing the confounding effects of other severe abdominal organ injuries. The frequency of adrenal gland injuries was 0.35%, with 1820 (0.3%) patients having adrenal contusions and 310 (0.05%) having adrenal lacerations. Compared to patients without adrenal gland injuries, those with adrenal gland injuries were younger and had higher injury severity scores. Obesity and severe abdominal trauma were more prevalent in patients with adrenal lacerations and contusions. The most common injury mechanism was motor vehicle accident. Patients with adrenal lacerations had a higher prevalence of severe injuries to the kidney, spleen, liver, pancreas, small intestine, colon, and rectum than those with adrenal contusions and those without adrenal gland injuries. Patients with lacerations of the adrenal gland had a significantly higher in-hospital mortality rate. Although adrenal gland lacerations are scarce, they are associated with severe injuries and higher fatality rates in adult trauma patients. Early recognition and prompt management of adrenal gland injuries can improve the outcomes in this vulnerable population.

## 1. Introduction

The adrenal glands, situated in the retroperitoneal area over the kidneys, play a vital role in the synthesis of various hormones.^[1,2]^ Traumatic injury to the adrenal glands can result in hemorrhage, contusion, or laceration.^[3]^ The adrenal glands are protected by the surrounding tissues, thereby reducing the risk of injury. Despite their rarity, with reported incidences ranging from 0.03% to 4.95% in previous studies,^[4,5]^ these injuries are associated with significant morbidity and mortality risks, especially in the context of severe multisystem trauma.

The existing literature on adrenal gland injuries in trauma patients is limited, with most studies being single-center retrospective reviews with small sample sizes.^[6,7]^ Consequently, the true incidence, associated factors, and outcomes of adrenal gland injuries after trauma remain poorly understood.^[4,6]^ The increased usage of computed tomography (CT) in patients has improved the early finding of adrenal gland lacerations, which were previously identified mainly during postmortem examinations.^[3,8,9]^ Previous studies have identified several factors associated with adrenal gland injuries in trauma patients, including high-energy mechanisms of injury, such as motor vehicle collisions and fall from height, as well as concomitant injuries to other abdominal organs, particularly the liver, and kidney.^[3,10,11]^

Studies have shown that patients who experience damage to both adrenal glands have severe injuries, an increased risk of complications, and an increased likelihood of mortality compared to patients with damage to only 1 adrenal gland^[12]^; however, some studies have suggested that adrenal gland injury is not a causative feature of mortalities,^[5]^ and that blunt adrenal injury (BAI) is not associated with poor outcomes.^[13]^ The impact of BAI on the prognosis of adult patients with isolated abdominal injuries was recently investigated by Duncan et al.^[14]^ Their research revealed a substantial correlation between BAI and higher severity of trauma in patients with polytrauma. However, they observed that mortality did not increase consistently among patients with adrenal injury, indicating that BAI may not autonomously affect the results. These results support the hypothesis that adrenal injury is associated with a generalized trauma burden, rather than functioning as a primary determinant of mortality.^[14]^ These authors arrived at the same conclusion in a pediatric population.^[15]^ The existing data has conflicting information about how adrenal injury affects patient outcomes; moreover, though various aspects of adrenal gland injury, such as laterality, polytrauma, and isolated injuries, have been discussed, there hasn’t been much emphasis on severe adrenal gland injuries like lacerations. These factors underscore the importance of conducting more focused studies on traumatic adrenal gland injuries of this nature.

To address this knowledge gap, we performed a large-scale national trauma data bank (NTDB) based investigation. The principal goals of this investigation were to ascertain the prevalence of adrenal gland laceration in adults who have experienced trauma, to identify the factors linked to the presence and severity of adrenal gland injuries, and to compare the results between patients who had and had not experienced adrenal gland laceration. Furthermore, the presence of concomitant abdominal organ injuries can confound the assessment of adrenal injury severity and associated outcomes. By excluding patients with severe liver, spleen, kidney, and gallbladder injuries, this study aimed to provide a more accurate understanding of the isolated effect of adrenal gland injuries on patient outcomes and hospital characteristics. The findings of this study could help improve our understanding of severe adrenal gland injuries in trauma patients and guide the development of evidence-based treatment strategies for this vulnerable population.

## 2. Methodology

### 2.1. Study design and data source

We used data from the NTDB, a large multicenter database that includes information on trauma patients. The NTDB contains data on patient demographics, trauma characteristics, clinical outcomes, and hospital course for patients who meet certain inclusion criteria, such as admission to a participating hospital for at least 24 hours or death following a traumatic injury.

### 2.2. Study population

Patients (≥18 years) who were admitted to the participating institutions with either blunt or penetrating traumatic injury in 2017 were included in our analysis. Patients transferred from another hospital, declared deceased upon arrival or died in the emergency department, and those with missing or incomplete data on critical variables of interest, including age, sex, injury severity score (ISS), injury mechanism, or adrenal gland injury status, were excluded. Patients with severe concomitant abdominal organ injuries, defined as liver, spleen, kidney, or gallbladder injuries greater than grade 2, were excluded to minimize confounding effects. Specifically, patients with grade III or higher kidney injuries, including cortical lacerations > 1 cm depth; lacerations extending through the renal cortex, medulla, and collecting system; completely shattered kidneys; and avulsed renal hila. Additionally, patients with liver injuries of grade III or higher were excluded, encompassing subcapsular hematomas > 50% surface area or expanding, lacerations > 3 cm depth, parenchymal disruption involving 25% to 75% of the hepatic lobe, and major hepatic vein injuries. Patients with spleen injuries of grade III or higher were also excluded, which included subcapsular hematomas > 50% surface area or expanding, lacerations > 3 cm depth, lacerations involving segmental or hilar vessels with devascularization > 25% of the spleen, and completely shattered spleens with hilar vascular injury. By excluding severe concomitant abdominal organ injuries, the study aimed to provide a more accurate assessment of the impact of adrenal gland injuries on patient outcomes and hospital characteristics. Adrenal gland injuries were classified according to the American Association for the Surgery of Trauma grading system as grade 1 (contusion) or 2 (laceration). The primary outcome was in-hospital mortality. Secondary outcomes included total intensive care unit (ICU) length of stay, total ventilator days, and complications, such as sepsis and organ/space surgical site infections.

### 2.3. Data collection and variables

We extracted data on patient demographics (sex, age, race), injury characteristics (mechanism of injury, ISS, abbreviated injury scale (AIS) scores for each body region), clinical outcomes (length of stay and in-hospital mortality), and hospital course (complications) from the NTDB using standardized data collection forms. We also collected data on the presence and severity of adrenal gland injuries, which were classified as contusions or lacerations.

### 2.4. Statistical analysis

The characteristics of the study population were summarized using descriptive statistics, which were stratified according to the presence and severity of adrenal gland injuries. Categorical variables were compared using the Chi-square test or Fisher exact test, as appropriate, and continuous variables were compared using the Kruskal–Wallis test. We employed multivariate logistic regression to identify factors that were independently associated with the presence of adrenal gland injuries, while accounting for potential confounders, including age, sex, mechanism of injury, and ISS. Multivariate logistic regression was also used to compare outcomes (in-hospital mortality, ICU admission, and complications) between patients with and without adrenal gland injuries after adjusting for potential confounders. All analyses were conducted using Stata/SE version 15.1 (StataCorp, College Station) and considered a 2-tailed *P*-value of <.05 to be statistically significant.

### 2.5. Ethical considerations

This study was approved by the institutional review board of the contributing NTDB institutions and a waiver of informed consent was granted due to its retrospective design and de-identified data. All data were stored on a secure server and were accessible only to authorized personnel.

## 3. Results

### 3.1. Patient demographics and injury characteristics

Among the 638,134 trauma patients included in this study, 1333 (0.2%) sustained adrenal gland injury. Patients with adrenal injuries were predominantly male (69.8% grade 1, 75.0% grade 2) compared to those without adrenal injuries (58.4%, *P* < .001). Adrenal injuries were more frequent in young adults aged 19 to 44 years (43.3% grade 1 and 55.0% grade 2) than in the no-injury group (37.0%, *P* < .001). Obesity was significantly associated with adrenal injury (43.3% grade 1 and 40.7% grade 2 vs 28.5% no injury, *P* < .001) (Table 1).

**Table 1:** Patient demographics and injury characteristics.

Characteristics	No adrenal gland injury (N = 636,801)	Contusion (N = 1193)	Laceration (N = 140)	*P*-value
Sex				
Male	371,638 (58.4%)	833 (69.8%)	105 (75.0%)	<.001
Female	265,052 (41.6%)	360 (30.2%)	35 (25.0%)	
Age group				
Young adults (19–44)	219,098 (37.0%)	513 (43.3%)	77 (55.0%)	<.001
Middle-aged adults (45–64)	164,436 (27.8%)	493 (41.6%)	52 (37.1%)	
Older adults (65+)	207,900 (35.2%)	179 (15.1%)	11 (7.9%)	
BMI group				
Underweight (<18.5)	25,362 (4.6%)	11 (1.0%)	2 (1.7%)	<.001
Normal (18.5–24.9)	196,020 (35.8%)	213 (20.0%)	27 (22.9%)	
Overweight (25.0–29.9)	170,255 (31.1%)	380 (35.6%)	41 (34.7%)	
Obese (≥30.0)	156,110 (28.5%)	462 (43.3%)	48 (40.7%)	
ISS group				
Minor (1–8)	322,821 (50.9%)	180 (15.1%)	13 (9.3%)	<.001
Moderate (9–15)	222,962 (35.1%)	326 (27.3%)	36 (25.7%)	
Severe (16–24)	56,646 (8.9%)	391 (32.8%)	46 (32.9%)	
Critical (25–75)	32,148 (5.1%)	296 (24.8%)	45 (32.1%)	
Abdomen/pelvis severity				
No injury	564,684 (88.8%)	1 (0.1%)	0 (0.0%)	<.001
Non-severe (AIS 1–3)	68,794 (10.8%)	1157 (97.0%)	129 (92.1%)	
Severe (AIS 4–6)	2699 (0.4%)	35 (2.9%)	11 (7.9%)	
Mechanism of injury				
Falls	303,328 (48.3%)	177 (15.1%)	15 (10.9%)	<.001
Motor vehicle traffic	176,587 (28.1%)	894 (76.2%)	97 (70.8%)	
Struck by/against	36,319 (5.8%)	10 (0.9%)	2 (1.5%)	
Firearm	26,663 (4.2%)	5 (0.4%)	8 (5.8%)	
Injury intent				
Unintentional	556,932 (87.8%)	1159 (97.5%)	131 (93.6%)	<.001
Self-inflicted	9733 (1.5%)	14 (1.2%)	0 (0.0%)	
Assault	62,770 (9.9%)	15 (1.3%)	9 (6.4%)	
Trauma type				
Blunt	559,467 (88.6%)	1178 (99.1%)	130 (92.9%)	<.001
Penetrating	57,273 (9.1%)	8 (0.7%)	9 (6.4%)	

AIS = abbreviated injury scale, BMI = body mass index, ISS = injury severity score

Injury severity, as measured using the ISS, was markedly higher in patients with adrenal trauma. Severe (ISS 16–24) and critical (ISS 25–75) injuries were observed in 57.6% of grade 1 and 65.0% of grade 2 adrenal injuries, respectively, compared to only 14.0% in the no-injury group (*P* < .001). Severe abdomen/pelvis AIS scores were significantly more common in the grade 1 (2.9%) and grade 2 (7.9%) adrenal injuries than in the noninjury cohort (0.4%, *P* < .001) (Table 1).

Motor vehicle traffic accidents were the predominant mechanism of adrenal gland trauma, accounting for 76.2% of grade 1 and 70.8% of grade 2 injuries (*P* < .001). Penetrating injuries were slightly less frequently associated with adrenal trauma (0.7% grade 1 and 6.4% grade 2) than in the no-injury group (9.1%, *P* < .001) (Table 1).

### 3.2. Concomitant abdominal organ injuries

Adrenal gland injuries were significantly associated with concomitant trauma to other key abdominal organs. Kidney injuries were present in 9.2% of grade 1 and 4.3% of grade 2 adrenal injuries, and only 0.3% in the no-adrenal injury group (*P* < .001) (Table 2). Similarly, splenic trauma was more common in patients with adrenal injuries (16.9% grade 1 and 18.6% grade 2) than in those without (1.5%, *P* < .001) (Table 2). Liver injuries were also more frequent in the adrenal injury cohort (23.3% grade 1, 35.7% grade 2 vs 1.2% no injury, *P* < .001) (Table 2).

**Table 2:** Concomitant abdominal organ injuries.

Injury type	No adrenal gland injury (N = 636,801)	Grade 1: contusion (N = 1193)	Grade 2: laceration (N = 140)	*P*-value
Kidney injury grade				
No injury	635,058 (99.7%)	1084 (90.9%)	134 (95.7%)	<.001
Grade I	1578 (0.2%)	108 (9.1%)	5 (3.6%)	
Grade II	165 (0.0%)	1 (0.1%)	1 (0.7%)	
Spleen injury grade				
No spleen injury	627,344 (98.5%)	992 (83.2%)	114 (81.4%)	<.001
Grade 1: minor/unspecified	5198 (0.8%)	125 (10.5%)	15 (10.7%)	
Grade 2: major contusion	4259 (0.7%)	76 (6.4%)	11 (7.9%)	
Liver injury grade				
No liver injury	629,452 (98.8%)	915 (76.7%)	90 (64.3%)	<.001
Grade 1: contusion	1198 (0.2%)	59 (4.9%)	3 (2.1%)	
Grade 2: minor/unspecified	6151 (1.0%)	219 (18.4%)	47 (33.6%)	
Pancreas injury				
No pancreatic injury	636,123 (99.9%)	1160 (97.2%)	135 (96.4%)	<.001
Grade 1: contusion	449 (0.1%)	28 (2.3%)	4 (2.9%)	
Grade 2: minor laceration	229 (0.0%)	5 (0.4%)	1 (0.7%)	

Patients with kidney injuries of grade III or higher were excluded, which included cortical lacerations > 1 cm depth, lacerations extending through the renal cortex, medulla, and collecting system, completely shattered kidneys, and avulsed renal hila. Additionally, patients with liver injuries of grade III or higher were excluded, encompassing subcapsular hematomas > 50% surface area or expanding, lacerations > 3 cm depth, parenchymal disruption involving 25% to 75% of the hepatic lobe, and major hepatic vein injuries. Patients with spleen injuries of grade III or higher were also excluded, which included subcapsular hematomas > 50% surface area or expanding, lacerations > 3 cm depth, lacerations involving segmental or hilar vessels with devascularization > 25% of the spleen, and completely shattered spleens with hilar vascular injury.

### 3.3. Clinical presentation and interventions

Patients with adrenal gland injuries presented with a significantly lower median systolic blood pressure (SBP) (134 mm Hg grade 1, 127 mm Hg grade 2 vs 140 mm Hg no injury, *P* < .001) and a higher median pulse rate (94 bpm grade 1, 102 bpm grade 2 vs 86 bpm no injury, *P* < .001). Severe Glasgow Coma Scale (GCS) scores were more prevalent in patients with adrenal injury (15.7% grade 1, 23.7% grade 2) than in those without adrenal trauma (4.7%, *P* < .001) (Table 3).

**Table 3:** Clinical presentation and interventions.

Characteristic	No adrenal gland injury (N = 636,801)	Grade 1: contusion (N = 1193)	Grade 2: laceration (N = 140)	*P*-value
SBP				
Median (IQR)	140.0 (124.0, 158.0)	134.0 (112.0, 150.0)	127.0 (107.0, 146.0)	<.001
Pulse rate				
Median (IQR)	86.0 (74.0, 99.0)	94.0 (80.0, 110.0)	102.0 (90.0, 115.0)	<0.001
Respiratory rate				
Median (IQR)	18.0 (16.0, 20.0)	20.0 (17.0, 23.0)	20.0 (18.0, 24.0)	<0.001
Total GCS				
Median (IQR)	15.0 (15.0, 15.0)	15.0 (14.0, 15.0)	15.0 (10.0, 15.0)	<.001
GCS severe	28,514 (4.7%)	184 (15.7%)	33 (23.7%)	<.001
Angiography				
None	17,653 (85.5%)	156 (72.2%)	32 (82.1%)	<.001
Angiogram only	1514 (7.3%)	17 (7.9%)	2 (5.1%)	
Angiogram with embolization	1468 (7.1%)	43 (19.9%)	5 (12.8%)	
Hemorrhage control surgery type				
None	11,722 (56.6%)	137 (62.8%)	15 (38.5%)	.006
Laparotomy	4286 (20.7%)	57 (26.1%)	14 (35.9%)	
Thoracotomy	1235 (6.0%)	9 (4.1%)	5 (12.8%)	
Extremity	1640 (7.9%)	8 (3.7%)	3 (7.7%)	

GCS = Glasgow Coma Scale, IQR = interquartile range, SBP = systolic blood pressure.

Angiography was performed more frequently in patients with adrenal injuries (27.8% grade 1 and 17.9% grade 2) than in the noninjury group (14.4%, *P* < .001). Angiography with embolization was markedly higher in the grade 1 adrenal injury cohort (19.9%) than in the no injury (7.1%) and grade 2 injury (5.1%) cohorts (*P* < .001) (Table 3).

### 3.4. Outcomes and hospital characteristics

Patients with adrenal gland injuries had significantly longer ICU stays than did those without adrenal trauma. The median ICU length of stay was 4.0 days for grade 1 and 3.0 days for grade 2 adrenal injuries, vs 3.0 days for the no-injury group (*P* < .001) (Table 4). In-hospital mortality was markedly higher in patients with adrenal injuries (6.5% grade 1 and 13.6% grade 2) than in those without adrenal trauma (2.6%, *P* < .001) (Table 4). Complications, such as sepsis (1.2% grade 1, 2.1% grade 2 vs 0.3%, no injury; *P* < .001) and organ/space surgical site infections (0.5% grade 1, 1.4% grade 2 vs 0.1% no injury, *P* < .001), were more common in patients with adrenal injuries (Table 4). Adrenal injuries were more frequently treated at university hospitals (55.2% grade 1 and 51.4% grade 2) than in the no-injury group (38.4%, *P* < .001) (Table 4).

**Table 4:** Outcomes and hospital characteristics.

Characteristic	No adrenal gland injury (N = 636,801)	Contusion (N = 1193)	Grade 2: laceration (N = 140)	*P*-value
Total ICU length of stay (d)				
Median (IQR)	3.0 (2.0, 5.0)	4.0 (2.0, 10.0)	3.0 (2.0, 11.0)	<.001
In-hospital mortality	16,417 (2.6%)	78 (6.5%)	19 (13.6%)	<.001
Hospital complication – sepsis	1605 (0.3%)	14 (1.2%)	3 (2.1%)	<.001
Teaching status				
Community	267,147 (42.1%)	375 (31.4%)	50 (35.7%)	<.001
Nonteaching	124,060 (19.5%)	160 (13.4%)	18 (12.9%)	
University	243,586 (38.4%)	658 (55.2%)	72 (51.4%)	
Verification level				
Not applicable	239,511 (53.0%)	583 (68.5%)	66 (67.3%)	<.001
Level I trauma center	157,885 (34.9%)	237 (27.8%)	28 (28.6%)	
Level II trauma center	54,433 (12.0%)	31 (3.6%)	4 (4.1%)	

ICU = intensive care unit, IQR = interquartile range.

### 3.5. Multivariable logistic regression analysis

After adjusting for age, GCS, SBP, and pulse rate, adrenal injury remained an independent predictor of in-hospital mortality. Compared to patients without adrenal trauma, those with grade 1 (odds ratio (OR) = 1.337, 95% confidence interval (CI) = 1.007–1.775, *P* = .045) and grade 2 (OR = 3.338, 95% CI = 1.756–6.345, *P* < .001) adrenal injuries had significantly higher odds of in-hospital death (Table 5).

**Table 5:** Multivariable logistic regression analysis for in-hospital mortality.

Variable	Odds ratio (95% CI)	*P*-value
Age (yr)	1.042 (1.041–1.043)	<.001
Total GCS	0.693 (0.690–0.695)	<.001
SBP	0.995 (0.994–0.995)	<.001
Pulse Rate	1.008 (1.007–1.009)	<.001
Adrenal injury grade (ref: no injury)		
Contusion	1.337 (1.007–1.775)	.045
Laceration	3.338 (1.756–6.345)	<.001

GCS = Glasgow Coma Scale, ISS = injury severity score, SBP = systolic blood pressure.

Reference groups are listed for categorical variables.

## 4. Discussion

Although rare, traumatic adrenal gland injuries are associated with high morbidity and mortality. This large-scale retrospective study using a well-established national trauma database provided a comprehensive analysis of the epidemiology, risk factors, and outcomes of adrenal gland injuries in this population of patients. In particular, this study is the first to focus specifically on adrenal gland lacerations and their impact on patient outcomes, in patients without any severe (greater than grade 2) liver, spleen, kidney, and gallbladder injuries to focus on the impact of adrenal gland injuries while minimizing the confounding effects of other severe abdominal organ injuries. The observed differences in adrenal gland injury patterns across the body mass index categories may reflect biomechanical factors. Retroperitoneal adipose tissue distribution could potentially influence trauma force transmission, although the exact mechanism remains unclear. Future biomechanical studies are needed to validate these hypotheses and elucidate the potential mechanical pathways. The differences in clinical presentation and physiological derangements among patients with adrenal laceration, adrenal contusion, and no adrenal gland injury provide valuable insights into the triage and management of these patients, with more severe derangements observed in patients with adrenal laceration, suggesting the need for a more tailored and quick response.^[16]^

Overall, adrenal gland injuries are rare, with varying incidence rates ranging from trauma patients.^[7,17]^ These injuries are often associated with severe trauma, as demonstrated by the high ISS, high frequency of hypovolemic shock, and need for surgical intervention.^[18–21]^ The wide range of previously reported adrenal injury rates highlights the need for a standardized method for the diagnosis and reporting of such injuries.^[13,17,20,22,23]^ Kunhivalappil et al, who investigated the incidence, associated injuries, and treatment of BAI found that the incidence of BAI was 0.72% among patients who underwent CT for blunt abdominal trauma, which is similar to the incidence reported in other studies. It is essential to recognize that the coexistence of concomitant abdominal organ injuries can complicate the evaluation of adrenal injury severity and its consequences. The identification of specific patient characteristics, severity, and mechanism of injury as risk factors for adrenal gland injuries offers valuable data for early recognition and management of these patients.^[10,13,24,25]^ By excluding patients with severe liver, spleen, kidney, and gallbladder injuries, our study provided a more precise understanding of the isolated effects of adrenal gland injuries on patient outcomes and hospital characteristics. Considering this fact, the prevalence of adrenal gland injury observed in our study appears to align with the aforementioned studies, which encompassed adrenal gland injuries along with severe liver, spleen, kidney, and gallbladder injuries.

Adrenal injuries are often associated with concomitant injuries to other organs, particularly the ribs, thorax, liver, and spleen.^[21,25]^ The presence of adrenal injury does not appear to increase mortality rates or prolong the hospital stay.^[22,26]^ Nevertheless, in patients with adrenal injuries, mortality was correlated with advanced age, higher ISS, chronic kidney disease, penetrating injuries, and injuries to the aorta/vena cava, peripheral vasculature, thorax, brain/spinal cord, and abdominal polytrauma.^[25]^ Isolated adrenal injuries are rare and not associated with increased mortality.^[27]^ Adrenal insufficiency is a potential complication of adrenal injury; however, it is not commonly documented, and few patients require chronic steroid therapy.^[17,18]^ Our study highlights the significance of considering adrenal gland injuries in patients without severe concomitant abdominal organ injuries, as these injuries were found to have a substantial relationship with in-hospital mortality. The differences in clinical presentation and physiological derangements among patients with adrenal laceration, adrenal contusion, and no adrenal gland injury provide valuable insights for the triage and management of these patients, with more severe derangements observed in patients with adrenal laceration, suggesting the need for a more tailored and rapid response. The identification of specific risk factors for adrenal gland injuries, particularly lacerations, can guide the development of targeted screening and diagnostic strategies for high-risk patients. The development of standardized protocols for the assessment and management of patients with suspected adrenal gland lacerations could help streamline care and reduce the variability in practice.

This study had several limitations that should be carefully considered when interpreting the results. As this was a retrospective study based on NTDB data, it may be subject to coding errors, missing data, or inconsistencies in data entry. The NTDB is a large multicenter database that delivers valuable information on trauma patients, but the quality and completeness of data may vary across participating institutions. The retrospective nature of the study also limits the skill to control for possible confounders or establish causal relationships between variables. Furthermore, the study focused on hospital outcomes and did not provide information on the long-term morbidity, mortality, or functional outcomes associated with adrenal gland injuries. Long-term follow-up data could provide additional insight into the impact of these injuries on patient outcomes. The study also lacked detailed information on injury mechanisms, laterality of adrenal gland injuries, and adrenal function, which could have provided further insight into the clinical implications of these injuries. Furthermore, the generalizability of the findings may be limited because the study was based on data from a single trauma registry, and differences in patient characteristics, injury patterns, and trauma care practices across different settings may influence the incidence, associated factors, and outcomes of adrenal gland injuries. Despite these limitations, this study offers valuable epidemiological data and emphasizes the significance of evaluating adrenal gland injuries in the context of patient characteristics, severity, and concomitant injuries in adult trauma victims.

The results of this study underscore the necessity of ongoing research to gain a more comprehensive understanding of the long-term repercussions of adrenal gland injuries in patients with trauma. Although the present investigation focused on in-hospital outcomes, these injuries may have enduring consequences for overall health, quality of life, and endocrine function. Longitudinal studies are required to assess the long-term outcomes of patients with adrenal gland injuries and identify factors that may affect these outcomes.

## 5. Conclusions

This study provides a comprehensive analysis of adrenal gland injuries in adult trauma victims without concomitant severe liver, spleen, kidney, or gallbladder injuries. The overall incidence of adrenal gland injury is 0.20%, with contusions being more common than lacerations. Significant differences in patient characteristics, injury severity, and associated injuries were observed among patients without adrenal gland injury, adrenal contusion, or adrenal laceration. Male patients, young adults, obese individuals, and those with a higher ISS and more severe abdominal trauma were more prevalent in the adrenal injury groups, particularly in lacerations. Patients with adrenal gland injuries, especially lacerations, had a higher prevalence of severe injuries to other abdominal organs and more severe physiological derangements. They also had longer ICU stays, more days on ventilators, higher in-hospital mortality, and a higher prevalence of hospital complications than those without adrenal injuries. Logistic regression analysis identified several factors associated with mortality, including adrenal injury grade, pulse rate, age group, SBP, severe GCS score, major mechanism of injury, kidney injury grade, and liver injury grade. These findings underscore the importance of prompt recognition and appropriate management of these injuries and the need for a thorough evaluation of concomitant injuries and close monitoring of potential complications. Further research with long-term follow-up and more detailed clinical data could help better understand the impact of adrenal gland injuries on patient outcomes and guide the development of evidence-based management strategies. Trauma teams should be aware of the potential for adrenal gland injuries in high-risk patients and should maintain a high index of suspicion to ensure optimal outcomes in this vulnerable population.

## Acknowledgments

The authors would like to thank the Deanship of Scientific Research at Majmaah University for supporting this work under project number no. R-2025-1597. The authors express their appreciation to the NTDB, its member hospitals, and the individuals who are responsible for the reporting and maintenance of the registry data. Statements Required for NTDB: The NTDB is the property of the American College of Surgeons, which maintains copyright ownership. The American College of Surgeons is not responsible for any claims that may result from derivative works that use original data, texts, tables, or figures. The authors also express their gratitude to the editors at www.editverse.com for their support in the areas of language editing and proofreading.

## Author contributions

**Conceptualization:** Musaed Rayzah.

**Data curation:** Musaed Rayzah.

**Formal analysis:** Musaed Rayzah.

**Investigation:** Musaed Rayzah.

**Methodology:** Musaed Rayzah.

**Validation:** Musaed Rayzah.

**Writing – original draft:** Musaed Rayzah.

**Writing – review & editing:** Musaed Rayzah.
